# Managing Fetal Ovarian Cysts: Clinical Experience with a Rare Disorder

**DOI:** 10.3390/medicina59040715

**Published:** 2023-04-06

**Authors:** Alina-Sinziana Melinte-Popescu, Radu-Florin Popa, Valeriu Harabor, Aurel Nechita, AnaMaria Harabor, Ana-Maria Adam, Ingrid-Andrada Vasilache, Marian Melinte-Popescu, Cristian Vaduva, Demetra Socolov

**Affiliations:** 1 Department of Mother and Newborn Care, Faculty of Medicine and Biological Sciences, ‘Ștefan cel Mare’ University, 720229 Suceava, Romania; 2 Department of Vascular Surgery, University of Medicine and Pharmacy “Grigore T. Popa”, 700111 Iasi, Romania; 3 Clinical and Surgical Department, Faculty of Medicine and Pharmacy, ‘Dunarea de Jos’ University, 800216 Galati, Romania; 4 Department of Obstetrics and Gynecology, University of Medicine and Pharmacy “Grigore T. Popa”, 700115 Iasi, Romania; 5 Department of Internal Medicine, Faculty of Medicine and Biological Sciences, ‘Ștefan cel Mare’ University, 720229 Suceava, Romania; 6 Department of Mother and Child Medicine, Faculty of Medicine, University of Medicine and Pharmacy, 200349 Craiova, Romania

**Keywords:** fetal ovarian cysts, ultrasound evaluation, complicated cysts, therapeutic approach

## Abstract

*Background and Objectives*: Fetal ovarian cysts (FOCs) are a very rare pathology that can be associated with maternal–fetal and neonatal complications. The aim of this study was to assess the influence of ultrasound characteristics on FOC evolution and therapeutic management. *Materials and Methods*: We included cases admitted to our perinatal tertiary center between August 2016 and December 2022 with a prenatal or postnatal ultrasound evaluation indicative of FOC. We retrospectively analyzed the pre- and postnatal medical records, sonographic findings, operation protocols, and pathology reports. *Results*: This study investigated 20 cases of FOCs, of which 17 (85%) were diagnosed prenatally and 3 (15%) postnatally. The mean size of prenatally diagnosed ovarian cysts was 34.64 ± 12.53 mm for simple ovarian cysts and 55.16 ± 21.01 mm for complex ovarian cysts (*p* = 0.01). The simple FOCs ≤ 4 cm underwent resorption (n = 7, 70%) or size reduction (n = 3, 30%) without complications. Only 1 simple FOC greater than 4 cm reduced its size during follow-up, while 2 cases (66.6%) were complicated with ovarian torsion. Complex ovarian cysts diagnosed prenatally underwent resorption in only 1 case (25%), reduced in size in 1 case (25%), and were complicated with ovarian torsion in 2 cases (50%). Moreover, 2 simple (66.6%) and 1 complex (33.3%) fetal ovarian cysts were postnatally diagnosed. All of these simple ovarian cysts had a maximum diameter of ≤4 cm, and all of them underwent size reduction. The complex ovarian cyst of 4 cm underwent resorption during follow-up. *Conclusions*: Symptomatic neonatal ovarian cysts, as well as those that grow in size during sonographic follow-up, are in danger of ovarian torsion and should be operated on. Complex cysts and large cysts (with >4 cm diameter) could be followed up unless they become symptomatic or increase in dimensions during serial ultrasounds.

## 1. Introduction

Ovarian cysts are one of the rarest gynecological disorders that develop during fetal life and could have important consequences both in utero and after birth. The estimated incidence of fetal ovarian cysts (FOCs) is cited in the literature as 1 in every 2500 live births [1]. FOCs typically manifest as functional or benign cysts, with the prevailing hypothesis regarding their etiology attributing their development to augmented levels of maternal estrogens, fetal gonadotropins, and placental human chorionic gonadotropin (HCG) in the fetus [2]. Newborn ovarian neoplasms, including cystadenomas, teratomas, and granulosa cell tumors, are rare occurrences [3].

A typical ultrasonographic presentation of a fetal ovarian cyst is that of an abdominal, usually anechoic, and thin-walled cyst, located superiorly and parasagittally to the bladder in a female fetus [4]. However, it is important to consider various differential diagnoses for FOC such as urachal cyst, mesenteric cyst, intestinal duplication anomalies, intestinal obstruction, and lymphangioma [5]. In rare cases, the identification of a “daughter cyst” serves as a distinctive sign of ovarian cyst presence [4,6].

FOCs are classified into two categories based on ultrasound (US) criteria, as established by Nussbaum: simple and complex [4]. A complex cyst is typically characterized by heterogeneity, the presence of hyperechogenic components, a thickened wall, free-floating material, fluid–fluid levels, and intracystic septations. Conversely, a simple cyst is typically described as an intrapelvic structure that is spherical, thin-walled, anechoic, unilocular, and less than 2 cm in diameter [4].

Fetal ovarian cysts typically exhibit stability throughout gestation and are known to dissolve spontaneously within the first few months following delivery. However, in rare instances, FOCs can lead to complications, such as torsion or bleeding during the prenatal period [7]. A retrospective study by Toker Kurtmen et al., which examined 28 cases of antenatal ovarian torsion, revealed that it can be misdiagnosed as a variety of cystic disorders, including intestinal duplication cyst/mesenteric cyst, complex ovarian cyst, mature cystic teratoma, and simple renal cyst [8]. Ovarian loss is commonly associated with torsion, which is often detected prenatally by alterations in the ultrasound image from a simple to a complex appearance, as well as an increase in size [9].

Following a diagnosis of fetal ovarian cysts, routine serial ultrasound examinations are necessary to monitor for any structural changes or complications such as massive hemorrhage, cyst rupture, or ovarian torsion resulting in infarction [10]. Large cysts may result in complications such as ascites due to the rupture of the cystic wall; mass effect; compression of ureters, inferior vena cava, and large and small intestines; intraabdominal adhesions; respiratory compromise; and ovarian torsion with or without autoamputation of the ovary [11,12]. To prevent such complications, some surgeons recommend prenatal aspiration of large fetal ovarian cysts, particularly for those greater than 4 cm in diameter.

A recent scoping review by Bucuri et al. reported a 34% higher risk of complications in cases of a complex fetal ovarian cyst diagnosis, based on an evaluation of 15 articles published within the last 10 years [13]. In addition, a systematic review and meta-analysis by Bascietto et al., based on 34 studies published up to 2017, showed a higher risk of ovarian torsion for cysts measuring ≥40 mm compared with those measuring less than 40 mm (odds ratio—OR: 30.8; 95% CI: 8.6–110.0) [7]. The authors also reported a recurrence rate of 37.9% (95% CI: 14.8–64.3%) for cases that underwent in utero aspiration, as well as a pooled proportion of 10.8% (95% CI: 4.4–19.7%) for ovarian torsion and 12.8% (95% CI: 3.8–26.0%) for intracystic hemorrhage after birth.

Pre- and postnatal management of fetal ovarian cysts is often a topic of debate among clinicians. Despite clinicians’ concerns regarding the safety of fetal cyst aspiration, Bagolan et al. [14] recommended prenatal cyst aspiration if the cyst diameter is greater than 5 cm or if the diameter increases by more than 1 cm per week. On the other hand, some authors suggest that there is no need for postnatal surgical treatment for simple ovarian cysts with the largest diameter of <5 cm or <4 cm [11,15].

Given the heterogeneous therapeutic approaches and rarity of this disorder, there is a need for more descriptive studies on FOC management to establish a comprehensive database for future meta-analyses. Therefore, the aim of this study was to evaluate the impact of ultrasound characteristics on the evolution and therapeutic management of FOCs.

## 2. Materials and Methods

This retrospective cohort study comprised a population of 20 pregnant women who were assessed at the Clinical Hospital of Obstetrics and Gynecology “Cuza-Voda” in Iasi, Romania, between August 2016 and December 2022, based on prenatal or postnatal ultrasound evaluations indicative of a fetal ovarian cyst. The Institutional Ethics Committee of Hospital “Cuza Voda”, Iasi, Romania (No. 790/25.01.2017) provided approval for the study, and all legal guardians of the newborns included in the study provided informed consent. All procedures were conducted in compliance with applicable regulations and guidelines.

We tested two hypotheses in accordance with the existing literature [9,15,16]. The first hypothesis postulates that simple cysts, small cysts (≤4 cm), and those that show a tendency to decrease in size during fetal life through regular follow-up, will regress and thus should be monitored in the postnatal period. The second hypothesis suggests that complex cysts, large cysts (>4 cm), or those with an increasing size during follow-up in fetal life, have a propensity for torsion with ovarian amputation, and thus necessitate surgical intervention in postnatal life [9,15,16].

The study included female fetuses with cystic masses in the lower abdomen, either unilateral or bilateral, that exhibited characteristics of simple or complicated ovarian cysts as per the Nussbaum categorization. Complicated ovarian cysts were defined as those having a fluid–debris level indicative of internal hemorrhage, septations with or without internal echoes, calcifications, or a solid component, in accordance with the literature [17]. Patients with urachal cysts, mesenteric cyst, intestinal duplication abnormalities, intestinal obstruction, and lymphangioma were excluded from the study, as well as those with incomplete medical records or whose mothers were unable to provide informed consent.

The present study retrospectively collected data on gestational age at diagnosis, sonographic characteristics of ovarian cystic masses, and pre- and postnatal care from the medical records of the patients. The admission diagnosis was confirmed using an obstetrical ultrasound examination, which was performed using an E8 scanner with a 4.8 MHz transabdominal probe (GE Medical Systems, Milwaukee, WI, USA), and the examination was completed by a vaginal approach using an intravaginal probe of 5–15 MHz when the fetal abdomen was accessible.

Periodic ultrasound scans were carried out until delivery to monitor the evolution of the cystic masses. Only cases with a high degree of confidence in the presumptive ultrasound diagnosis were included in the study, and no ultrasound-guided cyst aspiration was performed during pregnancy.

Postpartum ultrasound examinations were performed by neonatologists within 72 h of birth using a GE machine with a transabdominal 7.5 MHz transabdominal transducer (GE Medical Systems, Milwaukee, WI, USA). All neonates underwent examinations by neonatologists, radiologists, and pediatric surgeons after birth. Patients with simple FOCs that had the largest diameter of 4 cm or less were followed up postnatally with a transabdominal ultrasound examination at 6 months of age. Patients with large ovarian cysts (>4 cm) or complex structures were considered emergencies and referred to the pediatric surgery department for evaluation and surgical treatment.

**Figure 1:**
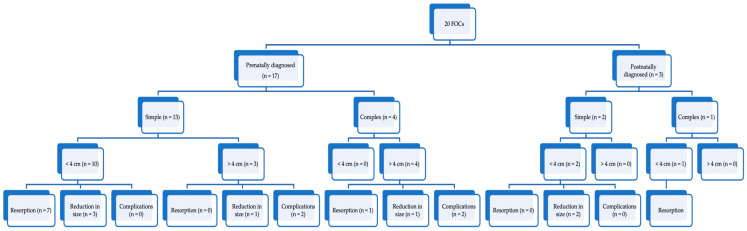
Flowchart of the evolution of FOCs in our cohort of patients.

The study recorded the ultrasound features and size of ovarian masses in neonates, with subsequent management consisting of sonographic follow-up or surgical intervention, including ovariectomy, cystectomy, or removal of an autoamputated ovary. No laparoscopic or ultrasound-guided aspiration was performed on neonates. Postnatal final diagnoses and outcomes were also documented. A perspective on the evolution of FOCs in our cohort of patients is presented in Figure 1.

The data were compiled into a database using SPSS software (version 28.0.1, IBM Corporation, Armonk, NY, USA), and the results were analyzed using descriptive statistics. Continuous data were expressed as means and standard deviations, while categorical data were reported as numbers and percentages. Each variable was evaluated with chi-squared and Fisher’s exact tests for categorical variables, and with t-tests for continuous variables.

## 3. Results

The present study investigated 20 cases of fetal ovarian cysts, with 17 cases (85%) being diagnosed prenatally and 3 cases (15%) postnatally. The prenatally diagnosed fetal ovarian cysts (FOCs) were classified as simple in 13 cases (76.48%) based on their ultrasound appearance, whereas 4 cases (23.52%) were deemed complex. Among the simple FOCs diagnosed prenatally, the majority (n = 10, 76.92%) had a maximum diameter of 4 cm or less, while only 3 cases (23.08%) had a larger diameter. Conversely, all complex FOCs (n = 4, 100%) diagnosed prenatally had a maximum diameter larger than 4 cm.

The mean gestational age at prenatal diagnosis and standard deviation were 26 ± 2.02 weeks for simple ovarian cysts and 32 ± 1.06 weeks for complex ovarian cysts. In terms of the size of prenatally diagnosed ovarian cysts, the mean size was 34.64 ± 12.53 mm for simple ovarian cysts and 55.16 ± 21.01 mm for complex ovarian cysts, achieving statistical significance (*p* = 0.01).

Regarding fetal ovarian cyst management, the simple FOCs with a maximum diameter of 4 cm or less, identified during prenatal and/or postnatal examinations, underwent resorption (n = 7, 70%) or size reduction (n = 3, 30%) without complications. However, only 1 simple FOC greater than 4 cm reduced in size during follow-up, while 2 cases (66.6%) were complicated with ovarian torsion. Complex ovarian cysts diagnosed prenatally underwent resorption in only 1 case (25%), reduced in size in 1 case (25%), and were complicated with ovarian torsion in 2 cases (50%).

No other fetal intra-abdominal malformations were identified during serial ultrasound evaluations, but two cases of ovarian cysts were associated with polyhydramnios. More images corresponding to the described cases can be found in the Supplementary Materials (Figures S1–S5). Only one case with a bilateral ovarian cyst was diagnosed and had a simple echogenic structure.

Moreover, 2 simple (66.6%) and 1 complex (33.3%) fetal ovarian cysts were postnatally diagnosed. All of these simple ovarian cysts had a maximum diameter of 4 cm or less, and all of them underwent a reduction in size. The mean diameter and standard deviation of the simple cysts diagnosed in the postpartum period (in the first 72 h of life) were 29.4 ± 10.5 mm. The complex ovarian cyst of 4 cm underwent resorption during follow-up.

Taken together, our cohort of patients comprised 15 simple (75%) and 5 complex (25%) FOCs. The demographic characteristics of the pregnant patients, fetal ovarian cysts’ location, and pregnancy outcomes are included in Table 1.

Patients diagnosed with simple fetal ovarian cysts were born from significantly younger mothers compared with those diagnosed with complex fetal ovarian cysts (*p* < 0.001). The majority of patients were born by cesarean section in both groups, but no statistically significant difference could be found between them regarding the type of birth.

The birthweight of newborns with complex ovarian cysts was significantly lower than the birthweight of those with simple FOCs (*p* = 0.01). However, we could not find any statistically significant difference between groups regarding their Apgar score at 1 min (*p* = 0.14) or the rate of preterm birth (*p* = 0.07).

After birth, patients with simple cysts <4 cm in diameter (5 cases, 25%) were referred for an ultrasound follow-up at 6 months. All simple cysts were reduced in size at the 6-month follow-up, with a mean diameter and standard deviation of 22.16 ± 0.98 mm. No other complications occurred. Meanwhile, patients with cysts with a diameter of >4 cm or with a complex structure (n = 4, 20%) were sent to surgery. Another two cases of FOC, which had an indication for surgery, underwent a conservative approach. The parents of 1 patient, with US features suggesting a fetal teratoma, opted for follow-up every 6 months, and during 3 follow-ups the cyst reduced its size to 4 cm. This patient underwent an MRI examination both prenatally and postnatally which confirmed the diagnosis of teratoma. Another patient had a complex cyst, but because of prematurity (delivery at 31 weeks), the surgical procedure was postponed, and the cyst disappeared in the next 4 months.

Two ovariectomies, one cystectomy, and a laparoscopic adnexectomy for the removal of an autoamputated ovary were performed on four patients with suspicion of ovarian torsion, which was further confirmed through surgery. During follow-up, all of these individuals were either symptomatic or had significant ovarian cyst growth. All surgical interventions were performed in the first weeks of life, and no complications occurred during or after surgery. Pathology reports indicated four hemorrhagic cysts.

## 4. Discussion

The aim of this study was to assess the influence of ultrasound characteristics on FOC evolution and therapeutic management. Our findings are consistent with previous studies examining various aspects related to FOC, thereby confirming our initial hypothesis.

The diagnosis of FOC is based on two non-specific ultrasound criteria: positive criteria, which involve the identification of a fluid-filled mass located in the lower and lateral region of the abdomen, above the bladder of a female fetus, and negative criteria, which involve a sonographic examination to confirm the integrity of the urinary and gastrointestinal tracts [18].

A pathognomonic sign for ovarian fetal cysts is the ‘Daughter Cyst Sign’, with a reported 82% sensitivity and 100% specificity [6]. It is a small, round, anechoic structure within a cyst, attached to its wall, and pathological studies demonstrated it was a follicle inside an ovarian cyst [6]. In our series, we observed this sign in 5 out of 17 cases diagnosed during prenatal sonographic examination.

Differential diagnosis is an important step in the prenatal and postnatal evaluation of fetal ovarian cysts, as many other abdominal or pelvic masses can mimic this pathology [19,20], and serological markers could be helpful in the postnatal differentiation of various types of masses [21,22]. Thus, we included only cases with highly suggestive ultrasound appearances of FOC in our study. The four surgeries performed in the postpartum period confirmed the cystic structures as belonging to the ovaries. No other associated structural abnormalities of the urinary or gastrointestinal tract were encountered during ultrasound examinations or during laparoscopic procedures.

Most fetal ovarian cysts are diagnosed during the third trimester. Chen et al. reported that 87.2% of fetal ovarian cysts were diagnosed after 28 weeks of gestation [9]. Moreover, Rotar et al. indicated in an observational study a mean gestational age of 31.28 weeks for the initial diagnosis of fetal ovarian cysts [23]. In our series, 8 cases (40%) were diagnosed during the second trimester, at the time of morphological ultrasound; 9 cases (45%) were diagnosed during the third trimester; and the remaining 3 cases (15%) were diagnosed after delivery, when an abdominal US was performed for clinical symptoms: abdominal enlargement in all cases, and tachycardia and vomiting in 2 cases.

The effectiveness of MRI in the diagnosis of prenatal and neonatal cysts is controversial. In contrast to the major contribution of MR imaging in fetal neoplasms, the clinical influence of MRI on the perinatal management of ovarian cysts may be limited [24]. Furthermore, when compared with the imaging of ovarian masses in pediatric and adult cohorts, where MRI can provide crucial information in confirming the diagnosis and selecting treatment, the importance of MRI in prenatal ovarian cyst management is minor [24]. In our series, an MRI was performed both prenatally and postnatally for a single case of complex neonatal cyst, suggesting a teratoma, because despite the physician’s recommendation of surgery, the parents opted for a follow-up, so we needed a second opinion regarding the diagnosis.

Most fetal ovarian cysts spontaneously regress during fetal life or during the neonatal period [25]. Others persist, and even increase in size, with a higher risk of complications such as ovarian torsion with the risk of autoamputation, intracystic hemorrhage, bowel obstruction, mass effect, or rupture, thus increasing the risk of surgical intervention, sometimes resulting in the loss of the gonad [26]. In our series, we had spontaneous resorption of FOC in 9 cases, the majority of whom (7 cases, 35%) were simple and had the largest diameter of 4 cm or less.

Regarding the prediction of complications and surgical need in FOC, considering cyst size, many authors support the following determinants of the neonatal outcome: a 4 cm cutoff, the US appearance (simple or complex), and the progression of the cysts during follow-up [7,27,28]. Small, unilocular cysts appear more likely to spontaneously resolve, whereas larger, complex cysts are at higher risk of persistence, complications, and surgical interventions [15].

Other authors recommend a cutoff of 50 mm for the cystic diameter to differentiate between cysts that spontaneously disappear and those at risk of complications [14,29]. In Bagolan’s statistics, 85% of ovarian cysts in neonates with a diameter of >50 mm required ovariectomy [14]. On the other hand, cysts less than 2 cm, and also very large cysts which, due to their volume, cannot be mobilized to twist, are almost never complicated by torsion.

In our series, all 4 cysts operated on for ovarian torsion had a diameter of > 4 cm. It is important to note that simple cysts, after torsion, increase in size and become complex in structure [30]. Moreover, the fluid–debris level seems to be a significant hallmark for ovarian torsion on ultrasound examination [17]. In our study, only two out of four cases of ovarian torsion were determined to be complex cysts. On the other hand, all cysts that suffered torsion increased in volume at serial US follow-ups.

Therapeutic approaches for ovarian fetal and neonatal cysts are still controversial. A recent study has shown that simple cysts smaller than 50 mm on postnatal imaging will likely spontaneously resolve and can be monitored using serial ultrasound examinations [29]. Due to the risk of bleeding, rupture, or intestinal blockage, several studies have recommended that neonatal cysts with complex characteristics be operated on as soon as possible [31,32,33]. Other publications, on the other hand, indicate a high rate of spontaneous remission of complicated cysts without complications, which we also found in our research [34,35].

Papic et al. found that postnatal torsion during observation is uncommon and that observation has no negative impact on the rate of ovarian preservation [29]. Given these findings, all asymptomatic neonatal ovarian cysts should be treated with surveillance. If surgery is required, an ovarian-preserving strategy should be used for all cysts, regardless of size, complexity, or the presence of prenatal or postnatal torsion, wherever possible [36].

We did not perform prenatal aspiration of the fetal cyst, which some authors have advocated because of the risks of preterm labor, chorioamnionitis, fetal injury, and fetal pain, as cited in the literature [36,37]. Furthermore, recurrence may occur due to persistent fetal exposure to hormonal stimulation after the procedure until birth [38]. The effectiveness of prenatal intrauterine aspiration in preventing neonatal surgery was compared with expectant treatment in a randomized open trial conducted by Diguisto et al. on 61 pregnant women whose fetuses were diagnosed with an anechoic ovarian cyst mass [36]. The authors reported that this procedure was associated with higher rates of in utero involution of cysts (47.1%; relative risk—RR: 2.54, 95%CI: 1.07–6.05), as well as reduced rates of oophorectomy after birth (3.0%; RR: 0.13, 95%CI: 0.02–1.03).

Our results support the conclusions of Dimitriaki et al. [39], suggesting that symptomatic neonatal ovarian cysts and those with increasing size at serial US follow-ups are at risk of torsion and must be operated on. For all ovarian cysts suspected of torsion, the diagnosis was confirmed intraoperatively, and one of the ovaries had already self-amputated at the time of surgery. Although all the cysts that became torsioned had a diameter of >4 cm, smaller dimensions than 4 cm cannot be considered a resorption criterion.

A multicentric retrospective study by Tyraskis et al. evaluated the risk of ovarian torsion in relationship to FOC size [40]. Their results showed that the rate of ovarian torsion increased from 0% in cysts measuring less than 20 mm to 33% in cysts measuring more than 50 mm, but they failed to demonstrate a statistically significant difference in this overall trend.

Our study has several limitations. First of all, this study has a retrospective design and includes a small number of patients. Furthermore, prenatal sonographic data on the size and changes in the appearance of the cyst throughout pregnancy were lacking for certain individuals, resulting in the diagnosis being made after birth in three patients.

Additionally, even if ultrasound imaging of the ovaries in the neonatal period can rule out autoamputation, the ovarian origin of the cyst, and even the diagnosis of hemorrhagic cyst without torsion, cannot be supported in cases where the cysts have been resorbed without surgical confirmation.

The strength of this study is the fact that it assessed the pre- and postnatal management of fetal ovarian cysts, which is a rare gynecological disorder, and our results could represent a base, along with other descriptive studies, for future meta-analysis that could offer clinicians a better assessment of the therapeutic strategies’ utility.

## 5. Conclusions

In conclusion, symptomatic neonatal ovarian cysts and those with increasing size at serial US follow-up are at risk of torsion and should be operated on. Complex cysts and large cysts (with a >4 cm diameter) could be followed up, unless they become symptomatic or increase in dimensions during serial ultrasounds.

The most important complication of fetal ovarian cysts is ovarian torsion, which represents a surgical emergency, and clinicians should be aware of this clinical scenario in the presence of large ovarian cysts with a complex ultrasound appearance and specific symptomatology.

FOCs can also have a malignant nature and should be carefully examined by a multidisciplinary team for specific symptoms, imaging features, and clinical risk factors in order to provide the best therapeutic management.

## Figures and Tables

**Table 1:** Clinical characteristics and ultrasound findings of FOC.

Parameter	Simple Cyst n = 15	Complex Cyst n = 5	*p* Value
Mother’s age (years)	26.1 ± 2.7	34 ± 2.1	<0.001
Gestation	1.9 ± 0.5	1.6 ± 1.1	0.40
Nulliparity (%)	Yes = 6 (40%)	Yes = 3 (60%)	0.43
GA at birth (weeks)	39 ± 0.36	37.2 ± 4.33	0.10
Type of birth (n/%)	Cesarean: 11 (73.3%) Vaginal: 4 (26.7%)	Cesarean: 3 (60%) Vaginal: 2 (40%)	0.57
Birthweight (g)	3374.54 ± 212.99	3080 ± 141.76	0.01
Apgar score at 1 min (mean ± SD)	8.64 ± 1.68	8.19 ± 1.93	0.14
Preterm birth (n/%)	Yes = 0%	Yes = 1 (20%)	0.07
Cyst location	Right: n = 10 (66.7%) Left: n = 4 (26.7%) Bilateral: n = 1 (6.7%)	Right: n = 4 (80%) Left: n = 1 (20%) Bilateral: n = 0 (0%)	0.78

Table legend: GA: gestational age; US: ultrasound.

## Data Availability

The data presented in this study are available on request from the corresponding author. The data are not publicly available due to local policies.

## Supplementary Materials

The following supporting information can be downloaded at: https://www.mdpi.com/article/10.3390/medicina59040715/s1, Figure S1: Ultrasound aspect of a simple fetal ovarian cyst. Figure S2: Bilateral simple fetal ovarian cysts. Figure S3: Complicated simple serous cyst. Figure S4: Fetal teratoma. Figure S5: Large fetal ovarian cyst.
