# Supplementary material for: Managing Fetal Ovarian Cysts: Clinical Experience with a Rare Disorder
Source: Medicina (Kaunas). 2023 Apr 6;59(4):715. doi: 10.3390/medicina59040715 (PMC10145213; doi:10.3390/medicina59040715)
Supplement: Supplementary file 1 [file medicina-59-00715-s001.zip › medicina-2324538-supplementary.pdf]

Supplementary materials

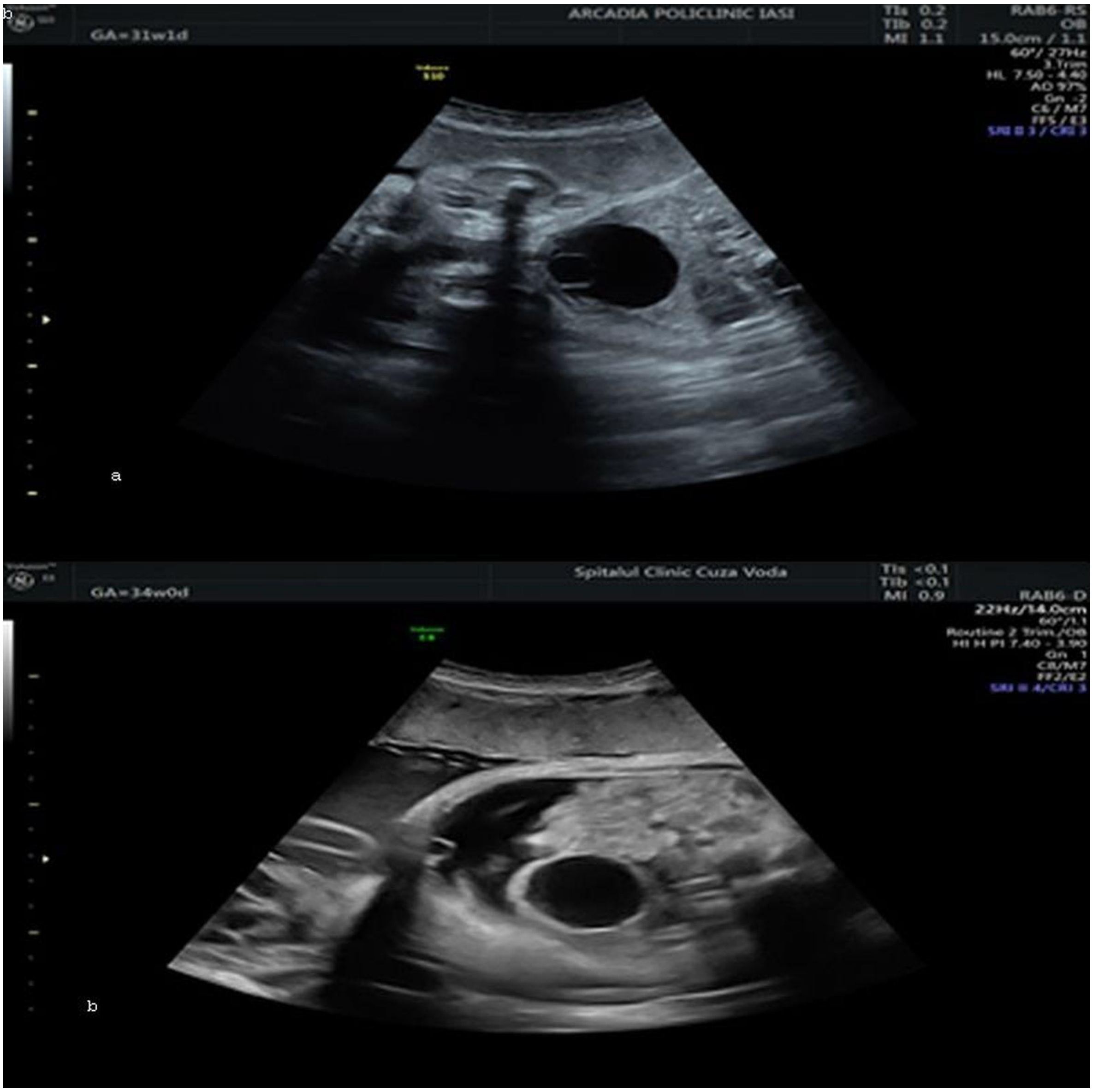

Figure S1. A 31-years old patient, IVG IIP, 31 weeks of gestation, simple serous cyst of 40mm-antepartum resorption. (**a**) Simple cyst with 40 mm diameter at 31w with daughter cyst sign; (**b**) At 33w rupture with fetal ascites.

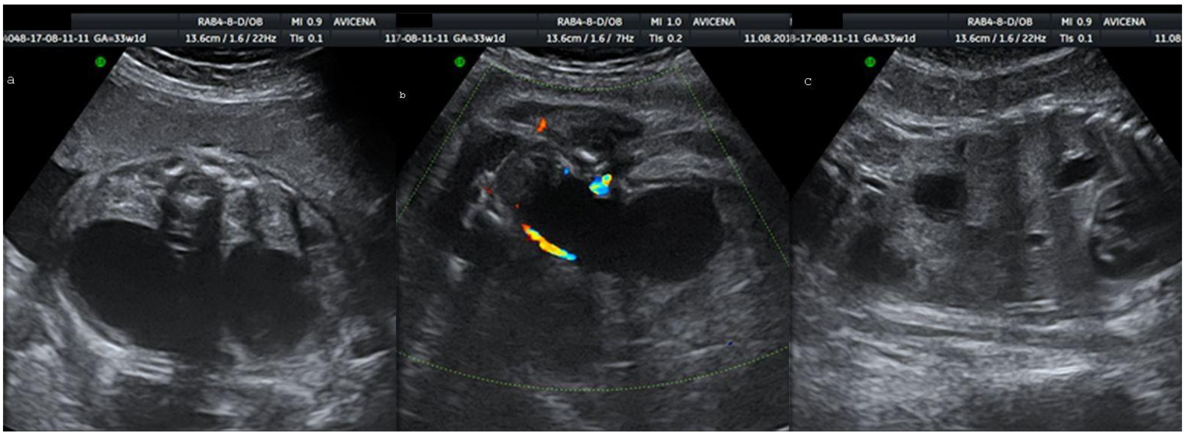

Figure S2. A 22-years old patient, IG IP, 33w. Size reduction in postpartum (**a**) bilateral simple ovarian cysts: right: 35,3mm; left: 38,5mm; (**b**) differential diagnostic with bladder; (**c**) Differential diagnostic with stomach.

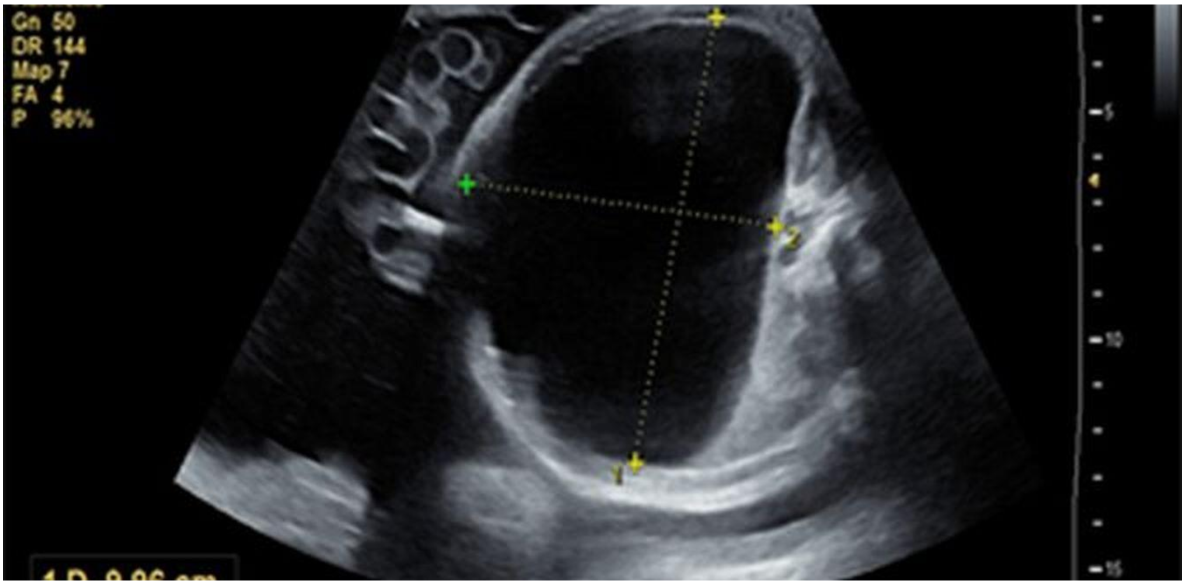

Figure S3. A 36-years old patient, IG IP 31weeks, serous simple cyst of 5,6/4.08cm; Evolution with increasing in volume until 10,16/8,54cm, with bowel compression and polyhydramnios (AFI=27cm); Growth and torsion. Ovariectomy for torsion.

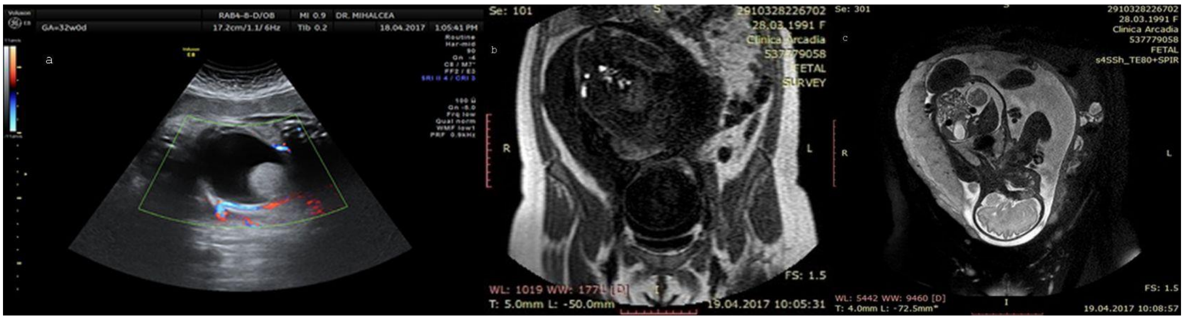

Figure S4. A 33-years old patient, IG IP, diagnosed at 33w with a fetal abdominal cystic mass of 70,4/58,3mm diameter, with a solid intracystic component of 29,2/30mm (ovarian teratoma or enteric cyst) In disagreement with the protocol, the family choose follow-up. At 6 months the tumor persists, but the dimensions are smaller: 32/32mm, and it keeps the same size at 1 year follow-up. (**a**) The ultrasound aspect; (**b**) The fetal MRI describe the cyst as teratoma of the ovary. T1 sequence; (**c**)The fetal MRI in T2 sequence.

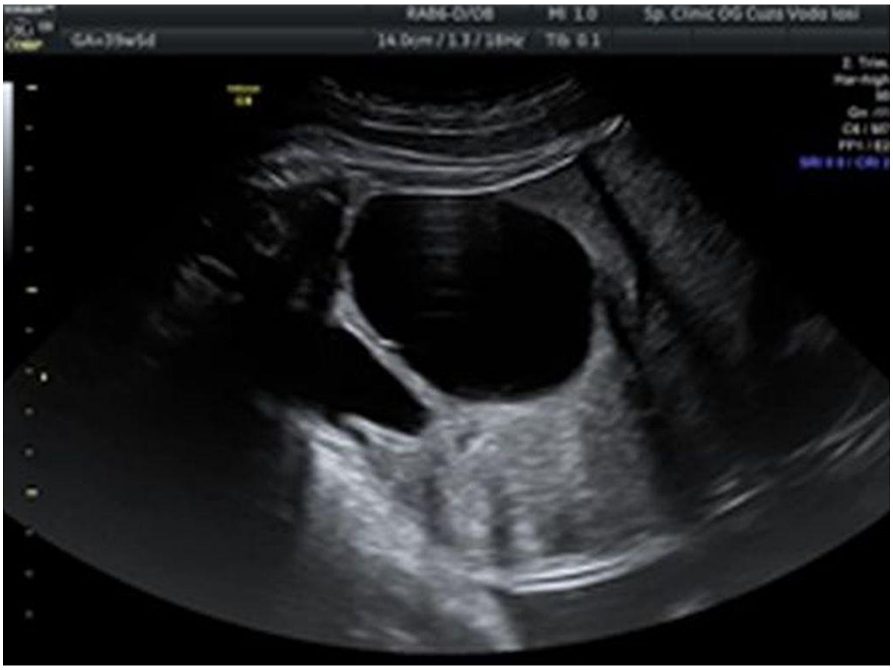

Figure S5. A 33-years old patient, IG IP, hospitalized for a fetal abdominal cyst of 6cm diameter. Cesarean delivery at 40w for anterior uterine scar (myomectomy). Stable general condition of the newborn. Pediatric surgery consultation at 2 weeks after birth and the decision is made for surgery due to the complex appearance of the cyst and the size that remains at 6-7 cm- ovariectomy performed for ovarian torsion with necrosis and intracystic hemorrhage .
